# Selenium and Selenoproteins: Mechanisms, Health Functions, and Emerging Applications

**DOI:** 10.3390/molecules30030437

**Published:** 2025-01-21

**Authors:** Shahidin, Yan Wang, Yilong Wu, Taixia Chen, Xiaoyun Wu, Wenjuan Yuan, Qiangqiang Zhu, Xuanjun Wang, Chengting Zi

**Affiliations:** 1 Key Laboratory of Pu-erh Tea Science, Ministry of Education, College of Food Science and Technology, Yunnan Agricultural University, Kunming 650201, China; shahidinwazir998@gmail.com (S.); wangyan2882@163.com (Y.W.); ynau_wuyl@126.com (Y.W.); 15870148875@163.com (T.C.); wuxiaoyun79@163.com (X.W.); yuanwj0805@126.com (W.Y.); tianjiao125@126.com (Q.Z.); 2 Research Center for Agricultural Chemistry, College of Science, Yunnan Agricultural University, Kunming 650201, China; 3 College of Resources, Environment, and Chemistry, Chuxiong Normal University, No. 546 S Rd. Lucheng, Chuxiong 675099, China

**Keywords:** selenium, selenoproteins, biosynthesis, structure insight, health functions, emerging applications

## Abstract

Selenium (Se) is an essential trace element crucial for human health that primarily functions as an immunonutrient. It is incorporated into polypeptides such as selenocysteine (SeC) and selenomethionine (SeMet), two key amino acids involved in various biochemical processes. All living organisms can convert inorganic Se into biologically active organic forms, with SeMet being the predominant form and a precursor for SeC production in humans and animals. The human genome encodes 25 selenoprotein genes, which incorporate low-molecular-weight Se compounds in the form of SeC. Organic Se, especially in the form of selenoproteins, is more efficiently absorbed than inorganic Se, driving the demand for selenoprotein-based health products, such as functional foods. Se-enriched functional foods offer a practical means of delivering bioavailable Se and are associated with enhanced antioxidant properties and various health benefits. Recent advancements in selenoprotein synthesis have improved our understanding of their roles in antioxidant defense, cancer prevention, immune regulation, anti-inflammation, hypoglycemia, cardiovascular health, Alzheimer’s disease, fertility, and COVID-19. This review highlights key selenoproteins and their biological functions, biosynthetic pathways, and emerging applications while highlighting the need for further research.

## 1. Introduction

**Figure 1:**
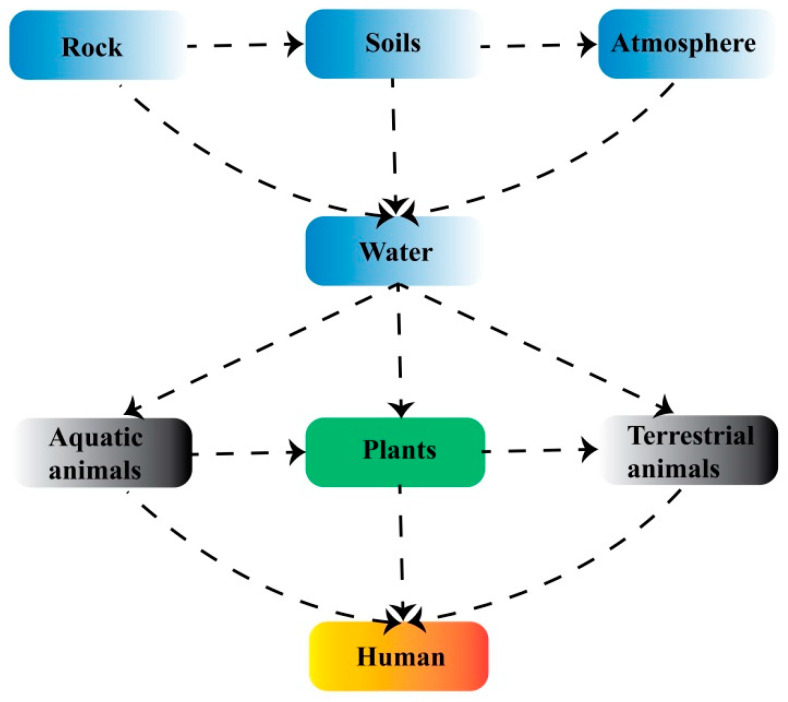
The primary routes through which selenium (Se) enters the human body are part of its biogeochemical cycle. This cycle starts with the weathering of Se-rich soils, rocks, and sediments, which releases Se into water sources. From there, it is absorbed by plants and animals through various mechanisms and, ultimately, makes its way into humans.

Selenium (Se) is an essential trace element that plays a significant role in maintaining human health and preventing disease [1]. Se deficiency can impair antioxidant defense systems, immune function, and thyroid hormone metabolism and contribute to disorders affecting neurological and endocrine functions and cardiovascular health as well as Keshan and Kashin–Beck diseases [2,3]. In the natural environment, Se primarily exists in two chemical forms: organic and inorganic. Inorganic Se further exists in different forms, including selenate (SeO_4_^2−^), selenite (SeO_3_^2−^), selenide (Se^−^), and elemental Se (Se^0^) [4]. Among these, SeO_4_^2−^ is the predominant inorganic Se compound found in nature. Organic Se is primarily represented by selenoamino acids, such as selenomethionine (SeMet) and selenocysteine (SeC), where Se substitutes sulfur (S) in the structures of methionine (Met) and cysteine (Cys), respectively. Additionally, methylselenocysteine (MeSeCys), which contains a methyl group along with Se replacing S, has been recognized as a major source of naturally occurring organic Se in plant-based foods for over a quarter of a century [5]. Figure 1 shows an overview of the transfer of Se from the environment to humans.

SeMet is the predominant Se compound initially identified in animals. However, Se’s most significant biological effects arise from its incorporation into an important class of proteins known as selenoproteins, which are present in the form of SeC. Selenoproteins perform diverse molecular roles and are involved in various biological functions and nutritional applications, unified by incorporating at least one SeC into their structure. SeC is recognized as the 21st naturally occurring proteinogenic amino acid. It differs from other amino acids by incorporating Se in place of S [6]. Genetic studies have revealed that the codon encoding SeC is uridine glycohydrolase activity (UGA). Functionally, SeC acts as both a structural and a functional analog of Cys, with the substitution of Se atoms for S enhancing its catalytic activity. According to studies, proteins containing SeC are classified as selenoproteins, while SeMet, which is not considered a selenoprotein, represents the non-specific utilization of Se [7]. Selenoproteins are found in archaea, bacteria, and eukaryotes in the form of SeC [8], derived directly from the diet or through the breakdown of SeMet. However, SeMet cannot be directly consumed, and it must be converted into SeC by being synthesized from the amino acid serine within the body. In plants, SeMet is primarily found in cereal grains, legumes, leafy vegetables, and soybeans, where it serves as the key precursor for the synthesis of SeC in animals [9]. In mammals, most identified selenoproteins are enzymes. Glutathione peroxidases (GPxs) were the first recognized selenoenzymes and play an important role in the organism’s antioxidant system [10]. These enzymes are essential in regulating lipid membrane oxidation, the NADPH-dependent reduction of thyroid hormones, thioredoxin (Trx) activity, and muscle metabolism [11]. Among the various selenoproteins, SeC is specifically incorporated into the active site via the UGA codon in mRNA [12]. Non-ruminant animals lack the ability to synthesize SeMet from inorganic Se sources such as SeO_3_^2−^ or SeO_4_^2−^. However, inorganic SeO_3_^2−^ and SeO_4_^2−^, along with SeMet, can be converted into SeC, present in the tissues of all animals. SeMet from the diet readily replaces methionine in muscle proteins, becoming an integral component of muscle structure and a reliable source of stored Se. In plants, selenoproteins are formed by incorporating Se-containing amino acids (SeC and SeMet) into proteins through a non-specific metabolic pathway involving S analogs, substituting Cys and Met. The 25 known selenoprotein genes in the human genome incorporate low-molecular-weight Se compounds in the form of SeC and play diverse roles in various biological functions [13]. Among the eight genes that encode glutathione peroxidases (GPxs), five (GPx1-4 and GPx6) contain SeC as the active-site residue. Additionally, there are three genes for thioredoxin reductases (TrxRs), three genes for deiodinases (DIOs), and one gene for selenophosphate synthase 2 (SPS2). The remaining selenoproteins include Sep15, SelH, SelI, SelK, SelM, SelN, SelO, SelP, SelR, SelS, SelT, SelV, and SelW [14].

## 2. Toxicity Levels and Recommended Daily Intake

The World Health Organization recommends a daily average intake of 55 µg of Se for adults, with intake recommendations varying by age, gender, diet, and geographic location [15]. The International Food and Nutrition Board suggests an average daily intake of 40–70 µg for men, 45–55 µg for women, and 25 µg for children [16,17]. Additionally, the lowest observed adverse effect level (LOAEL) for Se intake is approximately 4.3 μg/kg body weight per day (equivalent to 300 μg/day over five years), which is associated with increased mortality. The no observed adverse effect level (NOAEL) is 2.9 μg/kg body weight per day (around 200 μg/day), below which, no adverse effects, including increased mortality, have been observed [18].

## 3. Se Dynamics and Biosynthesis of Selenoproteins

The biosynthesis of selenoproteins involves the incorporation of Se into proteins, primarily as SeC or SeMet, both of which are essential for various biological functions. This process is dynamic and influenced by the chemical form of Se and the organism’s ability to metabolize it. While the fundamental mechanisms of selenoprotein biosynthesis are conserved across species, there are notable differences in how plants and eukaryotes process Se to form these functional proteins.

### 3.1. Biosynthesis in Plants

The uptake, translocation, and distribution of Se in plants are influenced by various factors, including plant species, developmental stages, physiological conditions such as salinity and soil pH, and the form and concentration of Se. Additionally, the activity of membrane transporters and the plant’s translocation mechanisms significantly influence Se dynamics within plants [19]. Plants primarily absorb Se in two forms: SeO_4_^2−^ and SeO_3_^2−^, although they can also take up organic Se compounds [20]. SeO_4_^2−^ is the predominant form of Se absorbed by plants, particularly in alkaline and well-oxidized soils, where it is more water-soluble than SeO_3_^2−^. Conversely, SeO_3_^2−^ is more bioavailable in anaerobic soils and wetlands [21]. Due to its chemical similarity to S, SeO_4_^2−^ is transported within plants using the sulfate transport system (SULTRs), while SeO_3_^2−^ is transported through the phosphate transporter system (PHTs) [22]. Upon SeO_4_^2−^ absorption, ATP sulfurylase (APS) catalyzes the hydrolysis of ATP to adenosine phosphoselenate (APSe), followed by reduction to SeO_3_^2−^ via APS reductase (APR) [23]. SeO_3_^2−^ is then converted to Se^2−^ by sulfite reductase and glutathione (GSH). The Se^2−^ is further converted into SeC in the presence of O-acetylserine (OAS) and OAS thiol lyase. Depending on environmental conditions, SeC may be methylated to methyl-selenocysteine (MeSeC) by SeC methyltransferase or transformed into SeMet. SeC is incorporated into proteins as selenoproteins. In non-hyperaccumulators, SeMet can be methylated to methyl-selenomethionine (MeSeMet), which is then converted to non-toxic volatile compounds like dimethylselenide (DMSe) or dimethyldiselenide (DMDSe) in hyperaccumulators [24].

### 3.2. Biosynthesis in Eukaryotes

**Figure 2:**
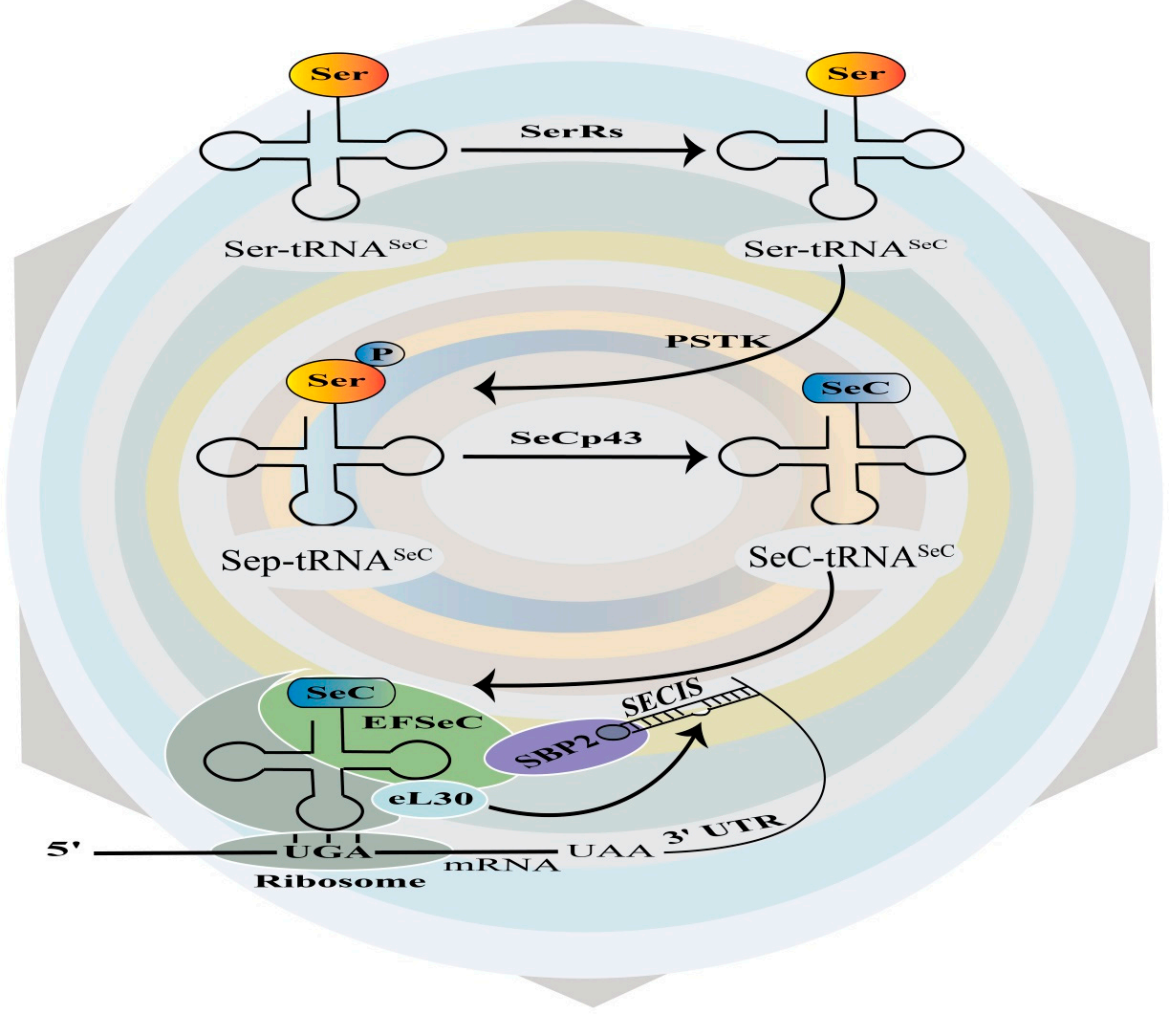
SeC incorporation and selenoprotein translation processes in eukaryotes.

Selenoprotein biosynthesis involves a unique mechanism in which the amino acid SeC is encoded by the UGA codon, which typically functions as a stop codon in standard mRNA translation [25]. This recoding process requires two essential RNA components. The first is the selenocysteine insertion sequence (SECIS), located in the 3′ untranslated region (UTR) of selenoprotein mRNAs. The SECIS element is crucial for UGA recoding, serving as a platform for assembling messenger ribonucleoprotein particles (mRNPs) that coordinate the recruitment of factors necessary for SeC insertion [26]. SECIS elements are characterized by conserved secondary structures, such as a stem-loop-stem-loop configuration, and contain motifs like the SECIS core and an AAA/G sequence, both essential for function [27]. The second component is SeC-tRNA^SeC^, a specialized tRNA that recognizes the UGA codon and facilitates SeC incorporation into the growing peptide chain [28]. Its biosynthesis starts with the charging of tRNA^SeC^ with serine by seryl-tRNA synthetase (SerRS) to form Ser-tRNA^SeC^. This intermediate is phosphorylated by phosphoseryl-tRNA kinase (PSTK) to produce Sep-tRNA^SeC^. Finally, selenocysteine synthase (SeCp43/SEPSECS) catalyzes the conversion of Sep-tRNA^SeC^ to SeC-tRNA^SeC^, the active form used during translation. Several proteins play important roles in selenoprotein synthesis. SECIS-binding protein 2 (SBP2) interacts with the SECIS element and facilitates the recoding of UGA as SeC. SBP2 contains RNA-binding and SeC insertion domains, enabling interactions with ribosomes and elongation factor EFSeC, which delivers SeC-tRNA^SeC^ to the ribosome [29]. Ribosomal protein L30 (eL30) also binds the SECIS element, stabilizing its structure and enhancing SeC insertion efficiency through interactions with SBP2 [30]. Additionally, 43-kDa RNA-binding protein may support selenoprotein mRNA processing, transport, and translation efficiency [31]. The efficiency of this biosynthetic pathway depends heavily on the functionality of the SECIS element, which regulates UGA recoding. Variations in SECIS structure or efficiency can affect selenoprotein expression, and mutations in SECIS have been linked to disorders like SEPN1-related myopathy [32]. Together, these components form a coordinated system for selenoprotein synthesis, enabling the integration of SeC into proteins with important biological functions (Figure 2).

## 4. Structural Insight into Mammalian Selenoproteins

Mammalian selenoproteins are categorized into two groups based on the position of SeC in their structure. The first group includes selenoproteins with SeC located near the C-terminus, such as TrxRs and selenoproteins K, R, O, I, and S. The second group comprises proteins with SeC positioned in the N-terminus region, including GPxs, DIOs, and selenoproteins H, M, N, T, V, W, Sep15, and SPS2. Many of these proteins share a Trx fold structure. Additionally, several selenoproteins in this group feature the CXXU motif, which corresponds to the Trx active-site CXXC motif, emphasizing their functional similarity [13]. This structural characteristic suggests that most selenoproteins are associated with redox-related reactions [33]. The most extensively studied families of selenoproteins are GPxs, TrxRs, DIOs, SELENOP, SELENOM, SELENOS, SELENOW, and SPS2. Although these selenoproteins perform distinct enzymatic functions, they all depend on reductants to supply the electrons required for their catalytic redox processes. Many characterized selenoproteins are enzymes that catalyze oxidation–reduction reactions, incorporating SeC within their active sites. Structurally, SeC is nearly identical to Cys, with the critical distinction being the presence of Se instead of S. Both Se and S belong to the same chemical family and share similar chemical properties. However, Se’s weaker outer valence electron bonding and inability to form various π bonds set it apart, enhancing its nucleophilic properties and enabling faster reactions with reactive oxygen species (ROS). While Se-oxide is more easily reduced than S-oxides, the Se-O bond lacks π-bond character [34]. However, SeC offers a functional advantage, as the thiol group of Cys is ionized less readily than the selenol group at physiological pH, rendering it more reactive in certain biological processes [35]. Substituting SeC with Cys in a selenoenzyme significantly reduces its catalytic activity. The structure and synthesis of mammalian SeC-specific transfer RNA (SeC-tRNA^SeC^) have been thoroughly reviewed in the recent literature [36]. Mammalian SeC-tRNA^SeC^ exists in two major isoforms, distinguished by the presence or absence of a methyl group on U34, the nucleotide in the wobble position of the anticodon [37]. Incorporating Se into proteins via plant accumulation induces notable changes in their structure and composition. Se integration affects secondary structural elements, such as the α-helix, β-sheet, and random coil. In selenoproteins, a significant proportion of Se is chemically bonded to proteins through S-S, Se-Se, Se-S, and thiol linkages [38]. The inclusion of Se can alter protein folding by replacing the C-S-S-C linkage of Cys with the C-Se-Se-C structure. This modification affects the secondary structure of the protein, disrupts disulfide bridges, and alters protein characteristics and functional properties. The tertiary structure of many proteins depends on disulfide bond formation, which occurs when two adjacent Cys residues undergo oxidation. In contrast, diselenide bonds, due to the larger size of Se atoms, are more stable than disulfide bonds and significantly lower redox potentials [39]. Among the 25 identified selenoproteins, a few significant ones are highlighted and discussed below.

### 4.1. Glutathione Peroxidases (GPxs)

**Figure 3:**
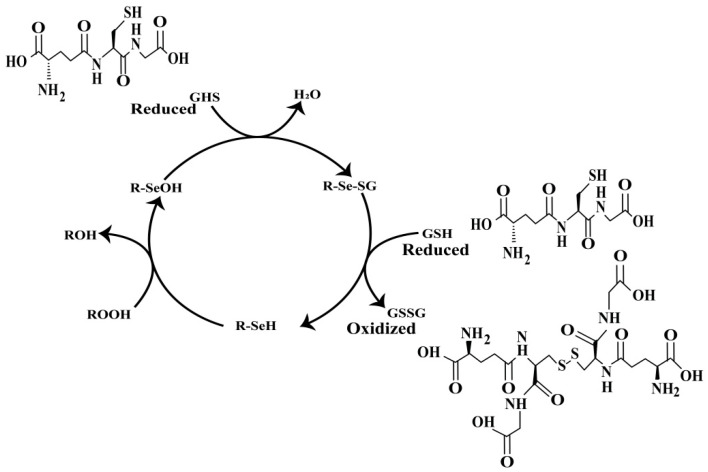
The catalytic cycle of GPx involves a series of steps. The R-SeH undergoes oxidation to R-SeOH. To restore its active form, selenic acid is subsequently reduced back to selenol by utilizing two molecules of GSH. The first GSH molecule forms a Se-GPx intermediate, producing water, while the second GSH molecule completes the reduction of the intermediate back to R-SeH, releasing GSSG. This cycle enables GPx to effectively neutralize oxidative damage caused by lipid peroxides and maintain cellular redox balance.

GPx1 was first identified by Mills in 1957 during his investigation into the mechanisms protecting erythrocytes from oxidative hemolysis [40]. Following Mills’ initial discovery, John Rotruck explored GPx1 in 1973 as part of his research into the antioxidant properties of Se [41]. Currently, eight members of the GPx family are known and designated GPx1 to GPx8. Among these, GPx1-GPx4 and GPx6 in mammals are selenoproteins, containing Se at their active sites as SeC. In contrast, the active sites of GPx5, GPx7, and GPx8 do not contain SeC but, instead, have Cys. GPx4 is unique in its ability to reduce complex lipid compounds, making it the only enzyme in the GPx family that directly reduces and eliminates lipid hydroperoxides [42]. GPx1 was identified as the first selenoenzyme in vertebrates. Since then, the GPxs family has expanded significantly, comprising a wide range of selenoenzymes involving the oxidation of selenolate to selenenic acid through a rapid, concerted reaction, where the selenolate nucleophilically attacks the hydroperoxide bond while an electrophilic proton stabilizes the reaction center. This unique mechanism eliminates the need for traditional substrate saturation, enabling GPx enzymes to neutralize hydroperoxides efficiently [10]. GPx1-3 are homotetrameric proteins, with each subunit having a molecular weight between 22 and 25 kDa, while GPx4 is a monomeric enzyme with a molecular weight ranging from 20 to 22 kDa. All well-known GPxs subunits exhibit a characteristic structural motif known as the Trx fold [43]. The defining feature of all GPxs enzymes is their ability to reduce hydroperoxides using thiols. Kinetic studies and enzyme–substrate interaction models suggest a reaction mechanism in which the selenol group (R-SeH) initially reacts with peroxides to form selenenic acid (R-SeOH). This is followed by a nucleophilic attack by GSH on the R-SeOH, leading to the formation of Se sulfide (R-Se-SG). The Se sulfide then reacts with another GSH molecule, regenerating the active selenol form of the enzyme [44] (Figure 3). This mechanism aligns with the distinct kinetics of GPxs, which do not follow the Michaelis–Menten model due to their highly efficient Se catalysis and flexible GSH binding. The enzyme’s surface-exposed Se promotes rapid reactions with H_2_O_2_, while the lack of a traditional binding pocket allows for diverse substrate interactions. These properties explain why GPx activity can be modeled using specific rate constants for the reduced enzyme’s reactions with ROOH and the subsequent reduction by GSH. This enzyme substitution or ping-pong mechanism highlights the enzyme’s efficiency and inability to form stable enzyme–substrate complexes, thus supporting the kinetic patterns observed experimentally [45].

### 4.2. Thioredoxin Reductases

**Figure 4:**
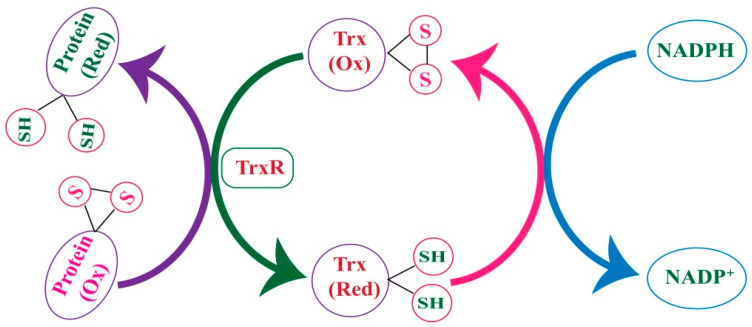
Trx redox system works by reduced Trx catalyzing the reduction of disulfide bonds (S-S) in oxidized cellular proteins, such as peroxiredoxin (Prx). As Trx undergoes oxidation during this process, it is subsequently reduced by TrxR, using NADPH as the electron donor.

Thioredoxin reductases (TrxRs) are enzymes from the pyridine nucleotide disulfide oxidoreductase family, with a molecular weight of approximately 12 kDa, and play a central role in reducing thioredoxin (Trx). Together, TrxR and Trx form a key antioxidant system that helps maintain cellular redox balance. This Trx system, which includes TrxR, Trx, and an electron donor in the form of NADPH, operates through a disulfide–dithiol exchange mechanism to regulate its antioxidant functions [46] (Figure 4). In mammals, three types of TrxRs have been identified. TrxR1 is located in the nucleus and cytoplasm [47], TrxR2 is found in mitochondria [48], and TrxR3 is found in the testis [46]. The entire Trx system relies on NADPH, driven by NADPH-dependent oxidoreductases, primarily cytosolic TrxR1 and mitochondrial TrxR2. Additionally, the system is Se-dependent because these enzymes are selenoproteins, meaning they contain a catalytic SeC residue. Moreover, these enzymes undergo splicing, producing different variants that alter their N-terminal regions.

### 4.3. Iodothyronine Deiodinases

**Figure 5:**
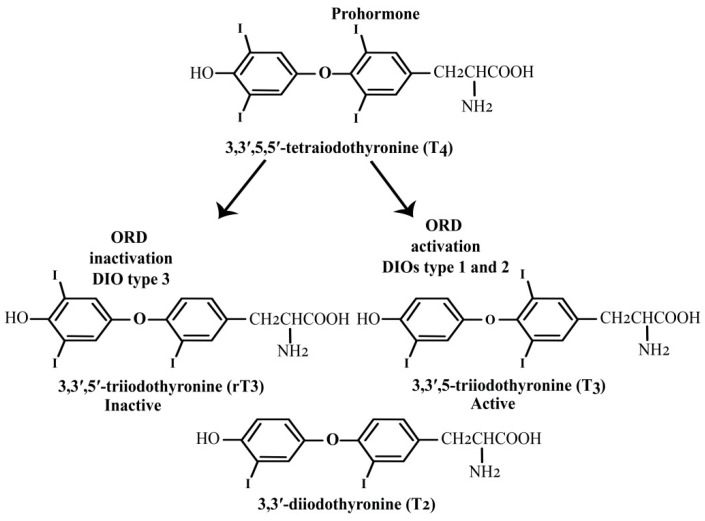
Structures and interactions among iodothyronine molecules that are either activated or inactivated by deiodinases.

Iodothyronine deiodinases (DIOs) are a family of enzymes essential for regulating thyroid hormone levels and metabolism in the body. There are three types of DIOs: DIO1, DIO2, and DIO3, each with a transmembrane domain and a catalytic center containing SeC, which facilitates the deiodination process [49]. These enzymes, with a molecular weight of approximately 60 kDa, are dimeric proteins containing a transmembrane domain. They are localized in different subcellular regions: DIO1 and DIO3 are found in the plasma membrane, while DIO2 is located in the endoplasmic reticulum, near the nucleus [50]. DIOs regulate the systemic and local availability of the thyroid prohormone thyroxine (L-thyroxine, 3,3′,5,5′tetraiodothyronine (T4), which is secreted by the thyroid gland. T4 is converted into the active form, 3,3′-5-triiodothyronine (T3), by the 5′-deiodinases DIO1 and DIO2 for outer ring deiodination (ORD) [51]. In contrast, DIO3, a 5-deiodinase selenoenzyme, inactivates both T4 and T3 and generates inactive reverse T3 (3,3′,5′-triiodothyronine, rT3) or 3,3′-diiodothyronine (3,3′-T2) by removing iodine from the inner ring of T4 or T3 through inner ring deiodination (IRD) [51,52], playing a key role in reducing thyroid hormone activity, especially during development and certain pathological conditions (Figure 5). The structural analysis of DIO enzymes reveals several important features critical to their function. Key structural motifs include the βαβ-motif, typical of thioredoxin-like folds, and a PRX signature motif (Ser/Thr-X-X-SeC/Cys), which is conserved across DIO enzymes. The DIO-insertion, a specific structural element found in DIO enzymes, forms a unique loop (Loop-D), helix (αD), and short strand (βD), which align with the central β-sheet of the thioredoxin-like fold. This insertion is important in defining the enzyme’s substrate-binding site, located between the conserved His202 and Arg275 [53]. These residues are crucial for substrate recognition, with His202 interacting with the phenol group of the substrate and Arg275 binding its carboxylate. Additionally, Sec170 (or Cys in mutants) plays a pivotal role in catalysis by enabling a nucleophilic attack on the substrate to form selenenyliodide. Other conserved residues, such as His219, Glu259, and Ser167, are involved in proton transfer through a hydrogen-bond network, which also involves Glu200, Tyr197, and Thr169, ultimately facilitating the catalytic process [54]. However, due to the lack of a dimeric structure, further structural studies with full-length, membrane-associated DIO enzymes are required to understand their function comprehensively.

### 4.4. SELENOP

**Figure 6:**
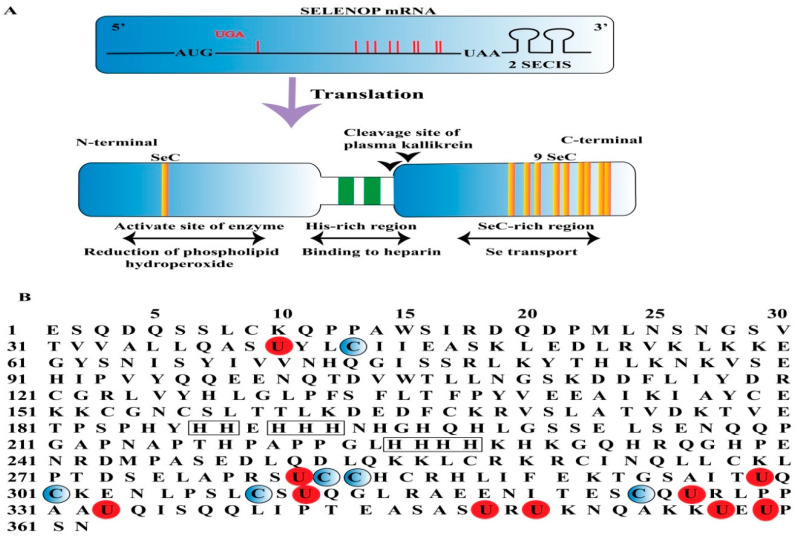
(**A**) The domain structure and function of SELENOP. (**B**) The amino acid sequence of human SELENOP. U (encircle red): SeC, C (encircle): Cys near SeC in the box where sequential histidine is displayed. Triangles show where plasma kallikrein cleaves.

Selenoprotein P (SELENOP) is unique due to its SeC-rich domain, which requires the incorporation of several SeC residues [55]. SELENOP, primarily synthesized in the liver, plays an essential role in Se transport to key tissues, including the endocrine glands and brain, where it helps maintain optimal Se levels [56]. Most identified selenoproteins contain one Se atom in the form of SeC per molecule; however, SELENOP is distinct in that it possesses 10 SeC residues in each polypeptide, all of which are encoded using the UGA stop codon, and it comprises half of the total Se content found in plasma [57]. Figure 6A illustrates two SECIS elements in the 3′ UTR of SeP mRNA [57]. The Se content in SeC within SELENOP is divided into two fragments [58]. In the N-terminal domain, one SeC is located at the 40th amino acid position, part of a UXXC redox motif. On the other hand, in humans, rats, and mice, the smaller C-terminal domain contains up to nine SeC residues [59]. Plasma kallikrein catalyzes the limited proteolysis of SELENOP, cleaving it at the Arg-235–Gln-236 and Arg-242–Asp-243 sites. The N-terminal fragment (residues 1–235) exhibits enzyme activity, while the C-terminal fragment (residues 243–361) demonstrates Se provision activity (Figure 6A) [60]. The human SELENOP amino acid sequence is shown in Figure 6B. The N-terminal region is proposed to function as a potential catalytic center of SELENOP characterized by a UXXC motif that closely resembles the active site configuration found in Trx (CXXC). This similarity suggests the possible reactivity of SELENOP with protein thiols. Furthermore, in the interaction between the C-terminal region of SELENOP and the YWTD β-propeller domain of the SELENOP receptor, ApoER2 has been recognized as significant, particularly concerning the regulation of Se levels in the brain and testes, as evidenced by the phenotypes observed in knockout (KO) mice [61]. Both SeC and Cys residues are abundant within the C-terminal region of human SELENOP (Figure 6B) and noted for their relatively high conservation across humans, mice, and rats [62]. Additionally, the middle portion of SELENOP contains a His-rich region featuring a conventional heparin-binding motif, characterized by the XBBXB pattern, where “B” represents a basic amino acid (Figure 6A). In its native state, SELENOP is a glycoprotein containing selenenylsulfide and disulfide bridges. It possesses three N-glycosylation sites at the N-terminal and one O-glycosylation site at the C-terminal, thus classifying it as a glycoprotein. Moreover, Se-supplemented HepG2 hepatoma cells also secrete N-glycosylated SELENOP [63]. These post-translational modifications are believed to impart specific structural characteristics to SELENOP and serve to protect Se by reducing its reactivity.

### 4.5. Others Selenoproteins

SELENOW is the smallest known selenoprotein in mammals, with a molecular weight of 9 kDa. It features a conserved Trx-like motif (CXXU) that contains a selenocysteine (SeC) residue [64]. It is predominantly expressed in the brain [65], muscle [66], and neurons and dendritic processes [67]. The structural interactions are between Trx1, SELENOW, and 14-3-3β, where Trx1 binds to the Cys32 residue in its CXXC motif and SELENOW and Trx1 bind to Cys191 of 14-3-3β. Both proteins compete for this binding site. In Trx1-deficient and SELENOW-deficient cells, increased subG1 population and PARP cleavage were observed after etoposide treatment, while Akt phosphorylation at Ser473 was reduced in Trx1-deficient cells but restored by SELENOW overexpression [68]. As a Se-dependent selenoprotein, SELENOW is abundant in the brains of both mice and humans [69]. The open reading frame (ORF) of the SELENOW gene consists of 393 base pairs (bp), encoding a 130-amino-acid protein. The 3′ untranslated region (UTR) is 372 bp long and contains the SECIS element. The SELENOW gene sequence in rainbow trout is longer than in mammals and most other fish species. The secondary structure of SELENOW is composed of a β1-α1-β^2−^β3-β4-α2 pattern [68].

Selenophosphate synthetases (SPS) utilize Se and ATP to produce selenophosphate, a key component for the biological use of Se, particularly in synthesizing SeC. As a result, SPS is essential for the proper functioning of selenoproteins, which are involved in various important processes, including protein quality control, redox homeostasis, hormone regulation, and metabolism. SPS is responsible for the production of selenophosphate, a highly reactive Se donor that is essential for the synthesis of Se-modified tRNA and the biosynthesis of selenoproteins [70]. This protein family consists of two subgroups, SelD/SPS2 and SPS1. The SelD/SPS2 subgroup represents the true SPS enzymes that are critical to Se metabolism and are found in all organisms that utilize Sec across the entire tree of life. Interestingly, many SelD/SPS2 proteins feature Sec as the catalytic residue within their N-terminal flexible Se-binding loop, while others substitute SeC with Cys [71]. SPS1 regulates cellular physiology, though it does not directly participate in SPS. It is expressed in rapidly proliferating cells and induced by oxidative and salinity stress. SPS1-deficient cells show inhibited cell proliferation, increased ROS, and disrupted redox balance. Its gene undergoes alternative splicing and its absence leads to phenotypic changes like embryonic lethality and impaired growth in model organisms [72].

SELENOM is an endoplasmic reticulum-resident thiol–disulfide oxidoreductase that is predominantly expressed in the brain and provides neuroprotective effects. Its active site features a Trx-like domain containing SeC, which is thought to facilitate thiol–disulfide exchange [73]. SELENOM regulates cellular redox balance and is crucial for leptin signaling in the hypothalamus. It enhances STAT3 phosphorylation and Ca^2+^ responses induced by leptin. Furthermore, SELENOM also has Trx activity, and its deficiency reduces Trx activity, leading to impaired cellular function. Additionally, SELENOM deficiency weakens the NF-κB pathway and increases vulnerability to ER stress-induced cell death [74].

SELENOS is a transmembrane protein of the endoplasmic reticulum that contains the rare amino acid SeC [75]. It is mainly recognized for its role in the endoplasmic reticulum-associated degradation (ERAD) pathway, which regulates the removal of misfolded proteins or improperly assembled protein complexes from the ER to the cytosol for proteasomal degradation. However, SELENOS also plays crucial roles in cellular signaling, including regulating transcription factors and cytokine levels [76].

## 5. Functions of Selenoproteins in Human Health

Due to their unique compositions and properties, selenoproteins are essential in maintaining human health. Some of these selenoproteins have well-characterized functions, as shown in Table 1.

Dietary Se, primarily incorporated into selenoproteins, is crucial for preventing health-related issues. The biological functions and nutritional benefits of Se are largely mediated through these selenoproteins. In humans, twenty-five genes encode selenoproteins, while, in mice, twenty-four genes are responsible for their production. Selenoproteins have been identified across all three domains of life, highlighting their widespread biological significance. The key biological functions of some important selenoproteins in human health are discussed below.

### 5.1. Antioxidant

**Figure 7:**
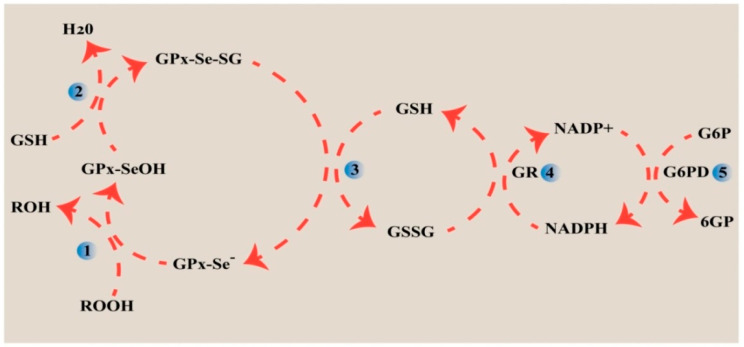
The pathway begins with the oxidation of selenol (-Se-) within GPx by peroxide (ROOH), forming selenic acid (-Se-OH). Next, a molecule of reduced GSH decreases -Se-OH to create a glutathionylated selenol intermediate (-Se-SG), releasing water in the process. Another GSH molecule further reduces the intermediate -Se-SG to produce GSSG, restoring GPX to its original selenol (-Se-) state. Subsequently, GSSG is converted back to reduced GSH by NADPH-dependent GR, with NADPH losing an electron to NADP+. Finally, glucose 6 phosphate (G6P), under the action of glucose 6 phosphate dehydrogenase (G6PD), reduces NADP+ to NADPH, completing the regeneration of NADPH essential for the reduction reactions catalyzed by GR.

Se is an essential trace element for mammals, widely recognized for its antioxidant properties. However, Se does not act independently as an antioxidant; rather, its biological function is largely attributed to its incorporation into selenoproteins, which play an important role in protecting cells from oxidative damage. Nearly half of the identified selenoproteins are associated with antioxidant defense mechanisms [110]. The Se-containing amino acid, SeC, is incorporated into the catalytic sites of these enzymes, where it participates in redox reactions and contributes to maintaining redox homeostasis and facilitates antioxidant defense and redox signaling [111]. Key families of enzymes that have been extensively studied in this context are the GPxs, which play an indispensable role in combating oxidative stress. GPxs catalyze the reduction of ROS, such as H_2_O_2_ and lipid H_2_O_2_, thereby mitigating oxidative damage [112]. Each member of the GPx family operates with distinct mechanisms of action and targets specific cellular sites to preserve redox balance. In mammals, GPx1-4 and GPx6 are classified as selenoproteins due to the incorporation of SeC in their active sites. These enzymes utilize SeC to reduce H_2_O_2_ and organic hydroperoxides, converting them into water or corresponding alcohols. This enzymatic activity is essential not only for mitigating oxidative damage but also for maintaining cellular integrity and proper function [42,113]. Interestingly, GPx6 is unique to humans among the GPx family members, adding another layer of specificity to Se’s role in human health. In contrast, GPx5, GPx7, and GPx8 rely on Cys residues at their active sites and thus do not function as selenoproteins. GPx5, for example, is predominantly expressed in epididymal tissue, which protects against oxidative stress in sperm cells [42]. GPx1 is an antioxidant using redox-sensitive SeC to reduce hydrogen peroxide and lipid hydroperoxides, with glutathione as the necessary electron donor. This reaction helps maintain cellular redox balance by modulating ROS levels [77]. In addition to the GPx family, the glutathione system comprising glutathione (GSH), glutathione reductase (GR), glutaredoxins (Grxs), and NADPH is a major thiol-dependent antioxidant mechanism that collaborates with selenoproteins to maintain cellular redox balance. Grxs, which possess a Trx-fold structure, are versatile proteins involved in redox signaling, iron metabolism, and antioxidant defense. They work in the cycle with GR to reduce oxidized glutathione (GSSG) back to its active form, GSH, thereby ensuring efficient cellular protection against oxidative damage (Figure 7). The GPx enzymes in particular interact with GSH and low-molecular-weight, soluble H_2_O_2_, but are less effective against more complex lipid H_2_O_2_ [10,114]. The TrxR-Trx pair is another important Se-containing enzyme essential in maintaining cell redox balance [46]. TrxR reduces Trx using NADPH, and Trx then reduces target proteins, modulating their function. In mammals, TrxR contains a SeC-Cys pair, while, in insects, it has a Cys-Cys pair. This system is essential for regulating processes like cell proliferation and apoptosis by controlling oxidative stress and redox signaling [115]. The Trx acts as an antioxidant by reducing ROS and preventing oxidative damage. Trx directly scavenges ROS by transferring electrons to oxidized proteins, reducing disulfide bonds, and restoring their functional conformation. TrxR recycles oxidized Trx using NADPH, ensuring a continuous supply of active Trx. This redox cycle helps maintain cellular homeostasis, protects against oxidative stress, and supports cellular processes like repair and survival under stress [116]. SELENOM is structurally close to Trx and shares similar redox-active properties. Like Trx, SELENOM contains a CXXU motif (instead of CXXC in Trx), which enables it to interact with and potentially regulate the Trx system. This structural similarity suggests that SELENOM may also participate in the antioxidant defense mechanism, particularly by modulating Trx activity and maintaining ROS homeostasis within cells. This antioxidant mechanism of SELENOM involves interacting with TXNIP (Trx-interacting protein) to regulate the Trx system. SELENOM reduces ROS levels by modulating Trx activity, inhibiting TXNIP expression. TXNIP is a negative regulator of the Trx system, and its upregulation leads to increased oxidative stress and apoptosis. SELENOM prevents this by maintaining Trx activity, thereby reducing ROS and protecting cells from oxidative damage. In neurons, SELENOM helps prevent neuronal apoptosis under oxidative stress by regulating cytosolic Ca^2+^ release from the ER [99]. Additionally, DIO enzymes responsible for the conversion of TH can influence oxidative stress indirectly. The activity of DIO1, DIO2, and DIO3 modulates levels of active T3, which can, in turn, affect metabolic processes that influence cellular redox status. DIO3 activity increases under oxidative stress, leading to the deactivation of T3, which may exacerbate tissue damage. Se, as well as antioxidants like N-acetylcysteine (NAC), can restore redox balance by reducing DIO3 activity and preserving T3 levels, thus improving cellular function under oxidative conditions [117]. Similarly, SELENOP plays a significant role in Se metabolism and overall antioxidant defense. SELENOP’s antioxidant function is attributed to its N-terminal SeC, located within a UXXC motif, which catalyzes the oxidation of GSH. In addition to its antioxidant role, SELENOP has been shown to influence steroid biosynthesis in Leydig cells, where it protects these cells from oxidative damage and supports testosterone production [56] SPS1 involves its role in ROS homeostasis and redox regulation in pluripotent stem cells. SPS1, through SeC, helps protect cells from oxidative stress by regulating ROS pathways and promoting cell survival. Knockdown of SPS1 alters ROS-related gene expression and increases apoptosis, while Se treatment improves cell survival in a dose-dependent manner, indicating the SPS1 role in SPS1-mediated antioxidant defense and redox signaling [118]. SELENOS reduces oxidative stress by regulating ROS levels and neutrophil extracellular trap (NET) formation. Silencing SelS increases ROS bursts, promotes NET formation, and enhances cytokine secretion, exacerbating inflammation and arteritis progression [119].

### 5.2. Anticancer Activity

The roles of both organic and inorganic forms of Se in cancer prevention are significant, with mechanisms including apoptosis induction, selective cytotoxicity, antiangiogenic effects, and the modulation of cell cycle progression [120]. A key factor in cancer cell survival is the excessive production of ROS due to elevated metabolic activity, which can damage cellular components. In response, cancer cells enhance their antioxidant defense systems by upregulating antioxidant proteins, thereby neutralizing ROS and promoting cancer progression [121]. Se plays an important role in this process through selenoproteins, which have been linked to various chemopreventive mechanisms, some well-established and others still under investigation. Selenoproteins, particularly GPx1, GPx3, TrxR, SELENOP, SELENOM, SELENOS, SPS1, and SPS2, are essential in regulating oxidative stress and cancer risk. Studies have demonstrated that the expression of selenoproteins correlates with reduced cancer incidence rates in lung, colon, and prostate cancers. The GPx1 gene, for example, acts as an anti-oncogene, and mutations in this gene are positively associated with increased cancer risk [122]. In animal models, the loss of both GPx1 and GPx2 results in ileocolitis and spontaneous cancer, underscoring the importance of these enzymes in cancer prevention [123]. Moreover, the anticancer mechanism of GPx2 in lung adenocarcinoma involves its role in maintaining cellular redox balance. By increasing antioxidant defenses, GPx2 helps cancer cells survive oxidative stress, contributing to tumor progression. High GPx2 expression supports tumor cell proliferation, invasion, and resistance to apoptosis. It is associated with more aggressive tumor characteristics (e.g., advanced stage, vascular invasion) and poor patient prognosis, making it a potential target for cancer therapy [124]. GPx3 is frequently hypermethylated in various cancers, and this downregulation is linked to poor prognosis and resistance to chemotherapy [125]. It has a dual role in cancer, acting as a tumot suppressor by preventing oxidative damage. However, in some tumors, it functions as a pro-survival factor, helping cancer cells resist oxidative stress. Se influences activity and cellular signaling [126]. Knockout models of GPx3 have revealed that its loss promotes cancer initiation, particularly when combined with genetic alterations or carcinogenesis models [127]. Furthermore, GPx4’s dual role in cancer is evident; though it acts as a tumor suppressor by inhibiting ferroptosis in certain cell lines, its overexpression can reduce the efficacy of cancer therapies, such as cisplatin [128]. The loss of GPx4 in Treg cells impairs immune tolerance and promotes antitumor immunity, suggesting that targeting Gpx4 could be a strategy to increase cancer treatment by modulating Treg cell function [129].

**Figure 8:**
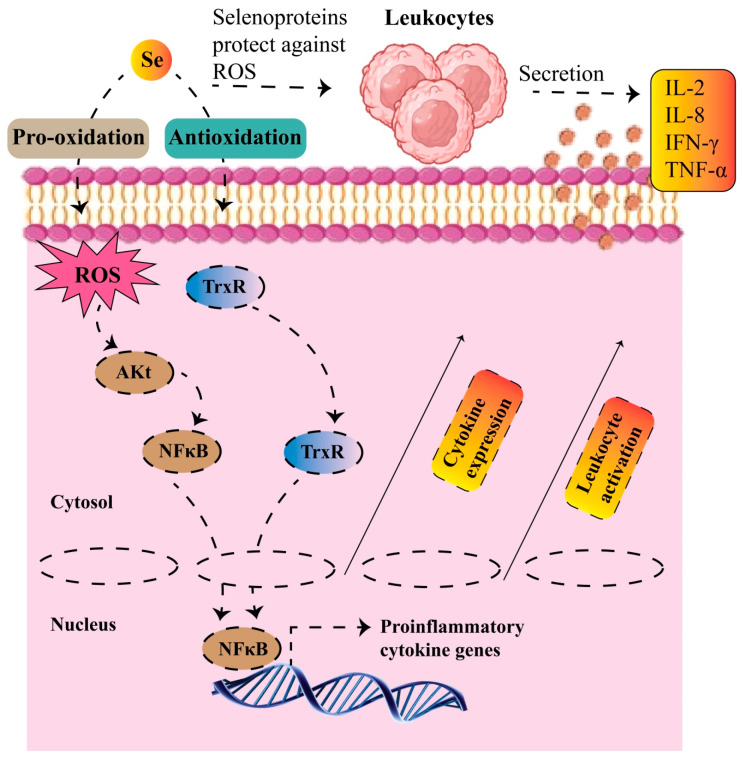
The proposed model demonstrates how Se influences the pro-inflammatory response in the tumor microenvironment and cancer cells. Se may act as a pro-oxidant, generating ROS and stimulating the Akt and NF-κB signaling pathway. Alternatively, it may function as an antioxidant by driving the synthesis of selenoproteins like TrxR, which relocates to the nucleus to activate NF-κB, thereby promoting the expression of pro-inflammatory cytokine genes and recruiting leukocytes. The proposed mechanism is adapted from [151].

The therapeutic targeting of TrxR has emerged as a promising strategy in cancer treatment. TrxR, which is overexpressed in many cancer types, is critical in regulating apoptosis and maintaining redox balance by transferring reducing equivalents to antioxidant enzymes [130,131]. TrxR inhibition disrupts this process, elevating oxidative stress within cancer cells, which are already vulnerable due to their high ROS levels. Furthermore, TrxR inhibition impacts DNA synthesis by limiting electron transfer to ribonucleotide reductase. The inhibition of TrxR has also been shown to induce apoptosis in cancer cells, suggesting that targeting this system could enhance therapeutic outcomes, especially in tumors with high oxidative stress [132]. Furthermore, when TrxR1 is inhibited, its inability to reduce Trx leads to increased oxidative stress, which is further exacerbated when the inhibited TrxR1 is converted into prooxidant SeCTRAPs. These SeCTRAPs amplify ROS production, contributing to cancer cell cytotoxicity and further supporting the potential of targeting TrxR for cancer therapy [133]. One study investigated the anticancer effects of thiazole compounds (T7 and T8) on glioblastoma (U-87 MG) cells. Both compounds reduced cell viability and induced apoptosis in U-87 MG cells, with T7 and T8 showing stronger effects than normal human dermal fibroblast (HDFa) cells. The compounds inhibited key redox-related enzymes, TrxR1, GST, and GR, critical for cancer cell survival. T7 selectively inhibited TrxR1 and GST, while T8 selectively targeted TrxR1 and GR. These enzyme inhibitions increase oxidative stress and promote cell death, making thiazole compounds promising candidates for glioblastoma treatment [134]. THs and their receptors also play critical roles in cancer cell behavior. Integrin αvβ3 on cancer cells provides insight into the biological function of iodothyronines. For instance, T4 stimulates angiogenesis and cell proliferation through αvβ3, independent of its prohormone role for T3 [135]. Although elevated T3 levels can promote cancer growth, maintaining normal T3 levels does not necessarily correlate with increased tumor growth in patients with low thyroid hormone levels [136]. The deiodinase enzymes (DIO1-3) regulate TH levels in tissues, and their dysregulation can impact carcinogenesis. Disruption of DIO function within tumors may significantly influence cancer progression [137]. Elevated DIO3 expression, in particular, has been implicated in several cancers, where it prevents differentiation by metabolizing THs into inactive forms, providing cancer cells with a survival advantage [138]. SELENOP, another Se-dependent protein, is pivotal in cancer progression and therapeutic responses. A decrease in SELENOP levels has been associated with resistance to chemoradiotherapy (CCRT) in cervical cancer, highlighting its potential as a modulator of cancer therapy sensitivity through lipid metabolism regulation [139]. In glioblastoma (GBM), SELENOP maintains GPx1 and GPx4 levels, contributing to ferroptosis sensitivity and offering a potential target for overcoming drug resistance [140]. It also plays an important antioxidant role in the development of colon cancer. Reduced levels of SELENOP may contribute to the onset of colorectal cancer [141]. Furthermore, low SELENOP levels are linked to an increased risk of developing cancers, including those of the kidney, colon, esophagus, and lungs [103]. SELENOP’s role across multiple cancers suggests its importance in cancer biology and prognosis [142]. The selenoprotein synthesis pathway is also targeted for therapeutic intervention. SPS2, for example, exploits the Se dependency of certain cancers, such as breast cancer, lymphoma, and melanoma by disrupting selenoprotein synthesis and inducing toxic selenide accumulation, which selectively targets cancer cells without affecting normal cells [71]. Similarly, SPS1 has been shown to modulate tumor immunity and DNA repair, with its expression correlating with immune cell infiltration and sensitivity to chemotherapeutics, such as 5-fluorouracil and Temozolomide [143]. SELENOS, another selenoprotein, influences cancer progression by regulating apoptosis and stress responses [75]. In colorectal cancer, SELENOS enhances ROS-induced stress and sensitizes cells to chemotherapeutic agents like regorafenib and oxaliplatin [144]. Similarly, in glioblastoma, the suppression of SELENOM triggers ER stress, leading to increased apoptosis and suggesting its potential as a therapeutic target [145]. It induces ER stress, activates the unfolded protein response (UPR) via the IRE1α signaling pathway, and depletes cellular Ca^2+^ stores. This disruption in Ca homeostasis triggers the increased expression of pro-apoptotic genes, leading to apoptosis in a dose-dependent manner. By promoting ER stress and disrupting calcium regulation, SELENOM enhances stress responses in cancer cells, positioning it as a potential anticancer agent [146]. The dual role of Se as both a pro-oxidant and an antioxidant has been widely debated. Se can act as either a pro-oxidant or an antioxidant within cells. For instance, Se deficiency can lead to oxidative stress due to decreased levels of selenoproteins like GPxs and TrxRs. On the other hand, excessive Se can cause a redox imbalance by oxidizing and cross-linking protein thiol groups, leading to increased ROS generation and cell death [147]. Generally, Se chemo-preventive effects are attributed to the antioxidant activity of selenoenzymes such as GPxs and TrxRs [148], with Se supplementation boosting the activity of GPx1 and GPx4 in humans. Additionally, higher dietary Se levels enhance the expression of certain selenoproteins. For example, T-lymphocytes from Se-supplemented mice showed increased GPx1 and TrxR1 activity [45]. Se status also correlates with the expression of inflammatory mediators, such as Akt, IL-1, NF-κB, and TNFα [149]. Since TrxR1 enhances the DNA-binding activity of NF-κB [150], it can be hypothesized that Se may activate NF-κB and related inflammatory pathways by boosting the activity of antioxidant selenoproteins (Figure 8).

### 5.3. Immune Enhancement

**Figure 9:**
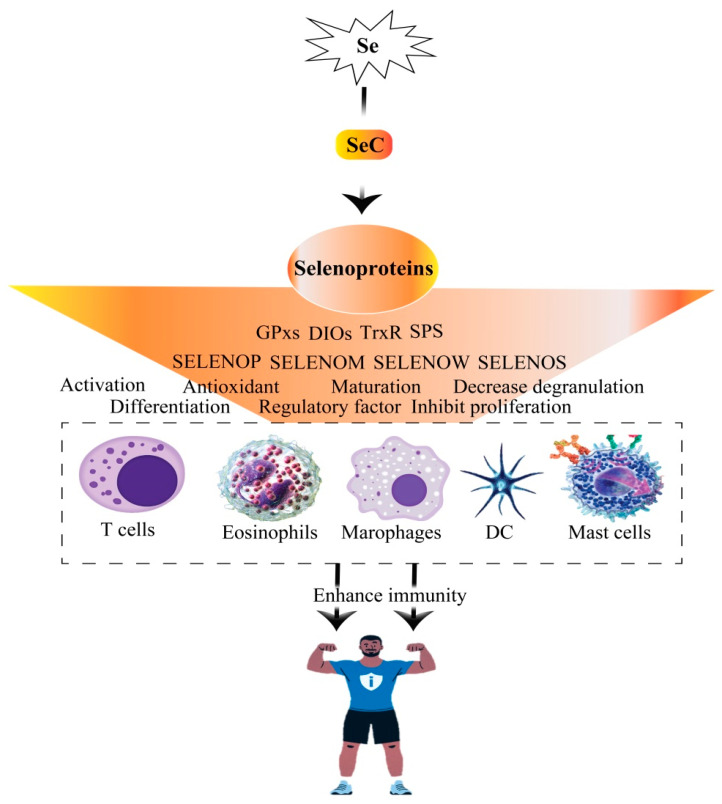
The figure illustrates how selenoproteins, derived from Se, regulate immune cells like T cells, eosinophils, macrophages, dendritic cells, and mast cells, enhancing immunity through activation, differentiation, maturation, regulatory factors, decreased degranulation, and antioxidant effects.

The biological function of Se in the immune system has been extensively studied, particularly its role in enhancing the production of selenoproteins. These proteins are integral to immune function, as the levels and form of dietary Se influence various immune system constituents. Adequate Se intake has been shown to enhance the expression of selenoproteins, which play a crucial role in the proper functioning of innate immune cells, including monocytes, macrophages, granulocytes, natural killer (NK) cells, eosinophils, mast cells, and dendritic cells (DCs). These cells form the body’s first defense against external invasion (Figure 9). These cells rely on rapid protective mechanisms, including the complement system, antimicrobial peptides, and enzymes, to target pathogens for lysis or phagocytosis [152]. A deficiency in Se can lead to impaired immune responses, increasing susceptibility to infections and potentially contributing to the development of cancer [153,154]. The GPx family of enzymes, including GPx1, GPx2, and GPx4, directly modulate inflammatory processes by regulating lipid mediators and the NF-κB signaling pathway. For example, GPx1 and GPx2 help control the production of pro-inflammatory prostaglandins by regulating COX-2, suggesting that these enzymes may have protective roles in inflammation-related diseases like colitis and cancer [155]. In autoimmune diseases like rheumatoid arthritis (RA), Se’s effect on GPx3 is critical. GPX3 neutralizes ROS produced by activated neutrophils in the synovial fluid, and the dysregulation of GPx3 exacerbates chronic inflammation and immune dysfunction, highlighting its potential as a therapeutic target in RA [156]. Moreover, Se’s role extends to regulating cellular processes like apoptosis and ferroptosis, which are tightly linked to immune cell homeostasis. GPx4, for example, modulates inflammation by eliminating oxidative species in the arachidonic acid (AA) pathway, thus preventing excessive inflammation by inhibiting the NF-κB pathway [157]. In chronic viral infections like hepatitis B, reduced GPx4 expression due to epigenetic alterations exacerbates oxidative stress, contributing to immune dysregulation and disease progression [158]. These findings underscore the importance of Se in maintaining oxidative balance and controlling inflammatory signaling in various immune-related diseases.

In addition to the GPx enzymes, thioredoxin reductase (TrxR) significantly modulates the immune response. TrxR regulates redox balance, crucial during inflammation, and influences inflammatory cytokine production by modulating the TLR4-NF-κB pathway. For instance, TrxR activation in corneal epithelial cells reduces the production of pro-inflammatory cytokines, indicating its role in controlling local inflammation [159]. In macrophages, TrxR inhibits the inflammasome pathway and promotes an anti-inflammatory response, further supporting its role as a critical modulator of immune function [160]. TrxR’s activity is tightly linked to Se intake, reinforcing the essential role of selenoproteins in regulating immune responses and maintaining cellular redox homeostasis during inflammation [161]. Butaselen, a TrxR inhibitor, enhances NK and T cell infiltration in HCC by upregulating CXCR3 and NKG2D. This improves immune cell function and responds to the tumor’s immunosuppressive microenvironment. When used alone, butaselen inhibits tumor growth and, in combination with PD-1 blockade, it synergistically enhances antitumor immunity, overcoming immune escape in HCC [162]. Recent studies have highlighted the important role of TH in regulating the function of innate immune cells. THs have emerged as key regulators of immune cell activity, influencing the body’s first line of defense against pathogens. The three deiodinases, enzymes responsible for activating or deactivating thyroid hormones, are expressed in various tissues but are not commonly found in immune cells. However, recent evidence suggests that innate immune cells, including neutrophils, macrophages, and dendritic cells, express thyroid hormone transporters, receptors, and deiodinase enzymes. These molecules modulate the availability of thyroid hormones in immune cells, which directly impacts their ability to recognize pathogens, initiate inflammation, and recruit other immune cells [163]. For instance, the deiodinase enzymes play a key role in converting thyroid hormones to their active or inactive forms, thereby influencing the immune response during infection. These also affect both innate and adaptive immune responses. By acting on immune cells, THs regulate local immune responses by modulating signaling pathways that control cell migration, cytokine production, and the differentiation of immune cell subsets. This regulation is essential for maintaining immune homeostasis and appropriately responding to pathogens. However, the full extent of the interaction between thyroid hormones and the immune system remains an active research area. It is known that THs can also influence systemic thyroid hormone levels by interacting with components of the immune system, potentially amplifying or suppressing immune responses depending on the thyroid status [164]. The transport of Se through SELENOP and its role in tissue distribution, particularly to the brain and endocrine glands, are important mechanisms that ensure proper Se utilization in immune cells and tissues. SELENOP’s role in Se homeostasis is critical during Se deficiency, as knockout mice lacking SELENOP experience severe symptoms, emphasizing the importance of this protein in sustaining adequate Se levels for immune function [56]. In experimental model systems, the paralog SECIS-binding protein 2-like (SECISBP2L) has recently demonstrated the ability to substitute for SECISBP2 in vivo [165], and it is necessary for thyroid hormone-dependent oligodendrocyte differentiation during development [166]. Analytical studies have shown that SELENOP can bind to heparin, indicating a potential role in protecting the endothelium and blood vessels.

As mentioned above, in its antioxidant and anticancer activity, SELENOM regulates the Trx system via its CXXU motif, reducing ROS by inhibiting TXNIP, a negative regulator of Trx. This action prevents oxidative stress and apoptosis, thereby maintaining cellular redox balance. In neurons, SELENOM further supports immune resilience by modulating ER Ca^2+^ release, indirectly supporting immune resilience [99]. Similarly, SPS1 plays a crucial role in maintaining redox balance in pluripotent stem cells by regulating ROS pathways through SeC, protecting cells from oxidative stress, and promoting survival. Knockdown of SPS1 disrupts ROS-related gene expression and increases apoptosis, while Se supplementation restores cell viability, emphasizing its role in antioxidant defense [118].

On the other hand, SPS2 targets the Se dependency of cancers such as breast cancer, lymphoma, and melanoma by disrupting selenoprotein synthesis and inducing toxic selenide accumulation, selectively eliminating cancer cells without harming normal tissues [71]. Studies also highlight the regulatory role of SELENOW in macrophages, where its deficiency disrupts redox balance, impairs anti-inflammatory pathways, and hinders the resolution of inflammation [167]. SELENOW’s interaction with key signaling pathways, such as EGFR-YAP1 in colitis models, further supports its role in regulating inflammation and tissue repair [168]. SELENOW’s role extends beyond immune regulation to bone metabolism, where its expression in response to inflammatory cytokines, such as TNFα, promotes osteoclastogenesis and bone loss in conditions like multiple myeloma [108]. In these models, SELENOW activates key transcription factors like NF-κB and NFATc1, driving osteoclast differentiation and bone destruction. These findings suggest that SELENOW’s role in immune regulation and inflammation is not limited to immune cells but extends to bone and tissue remodeling. Emerging evidence indicates that SELENOS is critical in glioma progression, specifically lower-grade gliomas (LGGs). SELENOS influences LGG progression by interacting with several upregulated proteins, positively correlating with SELENOS expression and LGG survival. In LGG cells, siRNA-mediated knockdown of SELENOS has been shown to reduce cell proliferation, viability, migration, and invasion while inducing apoptosis. Moreover, SELENOS expression is negatively correlated with temozolomide IC50, suggesting that it may affect drug sensitivity, further influencing immune cell infiltration within the tumor microenvironment [75].

### 5.4. Other Activities

Selenoproteins are implicated in various biological functions beyond their traditional functions, including involvement in metabolic disorders.

#### 5.4.1. Type 2 Diabetes

Although serum Se levels are not directly linked to the onset of type 2 diabetes, individuals with the condition often exhibit higher Se levels than those without. The precise relationship between Se status and insulin resistance, glucose metabolism, and the development of type 2 diabetes remains complex and not fully understood. Research indicates that Se influences insulin biosynthesis, secretion, and signaling through redox regulation by selenoenzymes [169]. High Se intake and elevated levels of selenoproteins can impair insulin signaling, leading to hyperglycemia and insulin resistance. However, the impact of low Se intake on glucose and carbohydrate metabolism is still unclear. Both hyperglycemia and hypoglycemia may occur due to Se deficiency, suggesting that balanced Se levels are necessary for proper glucose homeostasis [170]. In addition, specific studies have investigated the effects of plant extracts on glucose regulation. For example, the impact of bitter leaf extract on glucose levels and GPx activity in streptozotocin-induced type 2 diabetic rats showed reduced blood glucose without adverse effects on liver function [171]. However, the underlying mechanisms, such as how the extract influences oxidative stress or interacts with metabolic pathways, remain unexplained, and further research is needed to clarify the biochemical mechanisms involved. Additional insights into redox regulation come from studies on Trx, a redox-active protein. Trx1 was found to be released by MIN6 insulinoma cells under hypoxic conditions, and pancreatic islet grafts released Trx1 upon glucose stimulation. Supplementation with recombinant Trx1 countered hypoxia-induced apoptosis and preserved insulin secretion, suggesting that Trx1 activity supports β-cell function and survival under oxidative stress [172,173]. SELENOS, a selenoprotein, regulates hepatic lipid metabolism and insulin signaling. In SELENOS-deficient mice, hepatic steatosis developed due to increased fatty acid uptake and decreased fatty acid oxidation. This was accompanied by elevated ER stress markers and impaired insulin signaling, as evidenced by the reduced phosphorylation of IRS1 and Akt, contributing to insulin resistance.

Additionally, PKCε activation exacerbated insulin resistance in these mice. Conversely, the overexpression of SELENOS in hepatocytes reduced steatosis and improved insulin sensitivity [174], highlighting the importance of SELENOS in lipid metabolism and insulin signaling. These findings collectively point to a complex network where selenoproteins play key roles in regulating metabolic pathways involved in glucose and lipid metabolism. Their activities could influence the pathogenesis of type 2 diabetes and insulin resistance, emphasizing the need for further investigation into the precise molecular mechanisms involved.

#### 5.4.2. Male Fertility

Se plays a critical role in male fertility, particularly in the synthesis of testosterone and the development of spermatozoa. Selenoproteins, such as GPx1 and GPx3, are present in the male reproductive system, particularly in sperm and epididymal epithelia, protecting the developing sperm and surrounding tissues from oxidative damage [175]. One key selenoprotein, GPx4, phospholipid hydroperoxide glutathione peroxidase, is essential for safeguarding sperm cells from oxidative stress. Studies show that males with lower sperm GPx activity exhibit reduced sperm viability and motility [176]. The mitochondrial and sperm nuclear isoforms of GPx4 (mGPx4 and snGPx4) are integral to maintaining the structural integrity of mature sperm cells, highlighting their critical role in sperm function [177]. Another important selenoprotein in male fertility is TrxR3 (a component of the Trx/GR system, which is found in the testes). TrxR3 reduces both Trx and GSH, which is critical for maintaining redox balance in sperm. Deletion of the TrxR3 gene in mice leads to viable offspring but with impaired fertility, characterized by reduced fertilization rates and abnormal sperm motility. Proteomic studies indicate that TrxR3 is vital for regulating thiol redox processes essential for sperm quality and overall male reproductive function [178]. In addition, DIOs regulate TH production, and insufficient TH synthesis has been linked to various fertility issues, including irregular estrous cycles, implantation difficulties, and uterine abnormalities in both humans and rodents [176]. SELENOP plays a multifunctional role in male fertility, not only by reducing phospholipid hydroperoxides but also by facilitating the transport of Se from the liver to tissues such as the brain and testes. As a plasma protein, SELENOP is crucial for delivering Se to the testes, underscoring its importance in supporting male reproductive health [179]. SELENOS and SELENOW, along with other selenoproteins in mammalian systems, may contribute valuable insights into how Se and its associated proteins modulate male reproductive function. These findings suggest that Se plays a multifaceted role in male fertility through its action in redox regulation and sperm protection, while selenoproteins like GPx4, TrxR3, and SELENOP are crucial for maintaining sperm integrity and male reproductive health [180].

#### 5.4.3. Neuroprotection

Se is widely distributed in the body, with significant concentrations in the brain, where its levels remain relatively stable even during prolonged periods of low dietary intake. Alterations in Se levels have been observed in the brains and blood of individuals with brain tumors and Alzheimer’s disease (AD). Se exerts its neuroprotective effects in AD through the modulation of selenoproteins such as SELENOP, SELENOM, and SELENOS, as well as antioxidant enzymes like GPxs and TrxR. These selenoproteins play a crucial role in regulating key pathological features of AD, including the formation of amyloid beta (Aβ), tau phosphorylation, and neurofibrillary tangle formation. They also reduce oxidative stress and neuroinflammation, which are central to AD pathogenesis. Se supplementation has been shown to reduce Aβ aggregation, mitigate tau-related pathology, enhance synaptic plasticity, promote neurogenesis, and improve cognitive function in AD models. Se influences the gut microbiota by increasing beneficial bacteria, such as *Lactobacillus* and *Bifidobacterium*, which are linked to reduced AD pathology, suggesting an additional indirect mechanism through gut–brain interactions [181]. Through its selenoproteins, Se helps maintain brain homeostasis by neutralizing ROS, modulating Ca^2+^ channels, and regulating mitochondrial function. In AD, Se reduces amyloid β aggregation, prevents tau hyperphosphorylation, and protects against neuronal death, demonstrating its critical neuroprotective role [182]. One of the most abundant selenoproteins in the brain, GPx4, is a key regulator of ferroptosis, a form of iron-dependent cell death characterized by high lipid peroxidation levels [183]. Ferroptosis has been observed in brain regions, such as the hippocampus and forebrain, which are particularly affected in neurodegenerative diseases [184]. To counteract ferroptosis, enhancing GPx4 activity by increasing Se delivery to the brain is thought to help delay or prevent neuronal loss, making it a potential therapeutic target for neurodegenerative diseases like AD [185]. SELENOP, as a major Se transport protein, plays a significant role in maintaining brain function, especially in AD. It delivers Se to neurons, providing antioxidant protection, aiding cytoskeleton assembly, and interacting with redox-active metals like copper and iron. SELENOP also helps manage misfolded proteins, such as amyloid beta and tau, which are central to the pathology of AD. As the primary Se transporter in the brain, SELENOP is essential for neuronal health and may serve as a biomarker for Se status in neurodegenerative diseases [186]. Another selenoprotein, SELENOW, regulates tau homeostasis by promoting its ubiquitination through the ubiquitin-proteasome system. SELENOW competes with Hsp70 for tau binding, inhibiting tau acetylation at position K281 and enhancing tau degradation. In mice, SELENOW deficiency leads to tau dysregulation, synaptic defects, and memory impairments. Conversely, overexpression of SELENOW reduces tau-related pathologies, including tau phosphorylation, neurofibrillary tangle formation, and neuroinflammation, ultimately improving memory function in AD models [187]. Together, these findings suggest that Se, through its selenoproteins, plays a multifaceted role in protecting the brain from oxidative damage, regulating critical proteins involved in AD, and maintaining neuronal function. These mechanisms highlight the potential therapeutic value of Se in neurodegenerative diseases, particularly AD, by reducing the accumulation of toxic proteins, mitigating neuroinflammation, and promoting neuronal survival.

#### 5.4.4. Coronavirus Disease (COVID-19)

COVID-19, caused by acute respiratory syndrome Coronavirus 2 (SARS-CoV-2), shares similarities with its predecessor SARS-CoV. Se, through its selenoproteins, plays a protective role in reducing ROS, thereby combating oxidative stress during viral infections. Se helps mitigate inflammation by inhibiting the activation of NF-κB, a key regulator of immune responses. Additionally, selenoproteins are involved in modulating immune function by regulating type I interferon responses, controlling T cell proliferation, enhancing macrophage oxidative burst, and blocking viral transcriptional activators, thus providing a multifaceted defense against viral replication [188]. Epidemiological studies have highlighted the potential role of Se in COVID-19 outcomes. For example, a study in China found that regions with higher Se levels had better cure rates and lower mortality than regions with Se deficiency [189]. Similarly, a German study indicated that COVID-19 patients who survived had significantly higher Se levels, as determined by serum Se concentrations, than those who did not survive. Further investigation of Se status in COVID-19 patients revealed a significant deficiency in total serum Se and SELENOP concentrations compared to reference data from the large European EPIC study [190]. The research also assessed oxidative stress levels in COVID-19 patients, focusing on disease severity. The study involved 80 post-COVID-19 and 40 acutely ill patients, measuring Se levels, SELENOP, and oxidative stress markers such as malondialdehyde and 4-hydroxynonenal. The results showed that post-COVID-19 patients had lower, yet normal, Se levels, while acute patients had significantly reduced Se and SELENOP levels. Severe cases of COVID-19 were associated with higher oxidative stress, indicating increased free radical formation and an elevated need for antioxidant defense systems, such as those mediated by selenoproteins [191]. While Se intake appears to influence recovery rates, suggesting that selenoproteins may play a key role, the recovery beyond optimal Se intake indicates that other factors also contribute.

Furthermore, reduced expression of selenoproteins and elevated levels of inflammatory cytokine IL-6 in SARS-CoV-2-infected cells suggests a link between lower selenoprotein levels and COVID-19-related inflammation [192]. Research involving cultured Vero E6 cells has also shown that SARS-CoV-2 infection is associated with the decreased expression of critical selenoproteins, including GPx4 and TrxR3, which are important for maintaining cellular redox balance [193]. These findings emphasize that during COVID-19 infection, SARS-CoV-2 may disrupt the normal function of selenoproteins, exacerbating oxidative stress and inflammation and potentially contributing to disease severity. In summary, Se, through its role in modulating oxidative stress, inflammation, and immune responses, plays a critical protective role in COVID-19. The decreased expression of selenoproteins in infected cells highlights their importance in viral defense and suggests that Se supplementation could potentially support immune function and reduce inflammation in COVID-19 patients.

#### 5.4.5. Cardiovascular Diseases (CVDs)

CVDs represent a major health challenge, particularly in developed countries, with oxidative stress playing a central role in their initiation and progression. The excessive production of ROS contributes to DNA damage, lipid peroxidation, and protein oxidation and, ultimately, induces oxidative stress and cell death within cardiovascular tissues [190]. GPxs and TrxR, two important antioxidant enzymes, are crucial in mitigating oxidative damage and are significantly involved in the pathophysiology of CVDs [194]. One of the key selenoproteins, SELENOP, offers additional protection against CVDs by shielding vascular endothelial cells from oxidative injury. In populations with low levels of SELENOP, particularly in northern Europe, there is an increased risk of developing cardiovascular disease [195]. This underscores the importance of Se and its associated selenoproteins in cardiovascular health. Moreover, SELENOS (VIMP) could also affect cardiovascular function. SELENOS regulates cytokine production and the inflammatory response, which are critical in developing CVDs. In individuals with metabolic syndrome (MetS), reduced VIMP protein levels were observed, negatively correlated with waist circumference (WC) and positively correlated with HDL cholesterol levels. Additionally, VIMP expression showed a negative correlation with fasting blood sugar (FBS). The loss of VIMP expression may lead to the accumulation of misfolded proteins in the cytosol, impairing their function and disrupting various metabolic pathways, which could contribute to the development of CVD risk factors [196]. To improve overall cardiovascular risk, selenoproteins such as GPx3, SELENOP, and SeAlb influence disease outcomes. Research indicates that high total Se levels in selenoproteins, especially when coupled with low levels of GPx3, are associated with an increased risk of cardiovascular disease. This suggests that the redox-regulating functions of selenoproteins, particularly GPx3, are critical in controlling the oxidative stress and inflammation that drive cardiovascular pathology [197]. These conclusions highlight the importance of Se in cardiovascular health; while adequate Se and its selenoproteins are essential for protecting against oxidative damage and modulating inflammation, an imbalance, such as low GPx3 in the presence of high total Se, may contribute to heightened cardiovascular risk. The redox-regulating mechanisms of selenoproteins are thus crucial in determining cardiovascular disease outcomes. In CVDs, the Trx system regulates oxidative stress. TrxR reduces Trx using NADPH, activating Trx as an antioxidant. Elevated Trx levels are linked to conditions like heart failure and hypertension, where they help counteract oxidative damage. Conversely, Trx suppression leads to increased oxidative stress and worsened disease progression [198]. Additionally, recent studies have highlighted the role of GPx4 in mitigating ferroptosis in the heart. A proteomic study found that a decrease in GPx4 levels exacerbates ferroptosis during acute myocardial infarction (MI) [199]. Other studies support this finding, demonstrating that erastin, a known inducer of ferroptosis, decreases GPx4 expression in H9C2 cardiac cells [200]. Interestingly, using liproxstatin-1 to inhibit ferroptosis protected the heart from ischemic damage and helped restore GPx4 expression [201]. Studies have shown that selenoproteins associated with serum Se levels or having prognostic value play a crucial role in ferroptosis [202]. Recent studies suggest that targeting ferroptosis with strategies such as GSH supplementation, iron chelation, and other therapeutic approaches could offer promising treatments [203]. However, further research is needed to fully understand its mechanisms and potential for improving CVD outcomes.

## 6. Emerging Applications of Se-Enriched Functional Foods

Se is essential for human health, playing an important role in antioxidant defense, anticancer activity, and immune function through its incorporation into selenoproteins. Organic Se, in forms such as SeC and SeMet, is safer and better absorbed than inorganic forms. Current research emphasizes the development of Se-rich functional foods as safer, more bioavailable alternatives to pharmaceutical supplements, effectively addressing Se deficiencies while improving overall nutritional efficacy [204]. The dietary benefits of Se are increasingly evident in its incorporation into functional foods, including Se-enriched yeast, beer, kvass, and vegetables. These foods are particularly effective in combating Se deficiencies worldwide, ensuring adequate Se intake regardless of geographical location and providing regular consumption options for enhanced health [205]. Functional foods enriched with organic Se are considered excellent dietary supplements due to the high bioavailability of SeMet and other organic Se forms, which are beneficial for human nutrition [206]. Fortifying plants and animals to produce Se-enriched foods offers a practical solution for addressing Se deficiencies, especially in regions with low soil Se levels. This strategy is both safer and more effective than using inorganic Se, providing a reliable and accessible means of supplementation [207]. Natural food sources are particularly rich in Se, with Brazil nuts being among the richest, offering up to 512 µg/g of selenium [208]. Other foods, like broccoli, can accumulate Se when grown in Se-enriched environments [209]. Similarly, green tea and spirulina, capable of Se enrichment, also provide health benefits [210,211]. Certain edible fungi, such as *Grifola frondosa* and *Hypsizygus marmoreusalso*, absorb and accumulate Se [212,213].

Advances in food technology have enabled the production of Se-enriched products, such as Se-enriched yeast, wheat, and fermented foods like sourdough, all of which provide bioavailable forms of Se, including SeMet. These Se-enriched foods ensure safety, efficacy, and long-term usability [214]. Se-enriched yeast and SeMet are particularly effective for dietary supplementation, creating a reservoir of bioavailable Se with low toxicity. While inorganic Se compounds like SeO_3_^2−^ may be used for immediate therapeutic effects, Se-enriched foods are ideal for long-term, population-wide supplementation [11]. Finland’s pioneering work with Se-enriched fertilizers exemplifies the potential for improving Se nutrition in deficient regions. This approach underscores the importance of monitoring supplementation processes to ensure consistent safety and product quality [204].

In conclusion, Se-enriched functional foods derived from plants, yeast, and animal-based products effectively address Se deficiencies. These foods not only enhance Se intake but also improve overall health by providing safe and bioavailable supplementation. Advances in food science and technology continue to optimize the safety, quality, and accessibility of Se-enriched products, making them a cornerstone in the global effort to combat Se deficiencies [215].

## 7. Conclusions and Future Prospectives

Se, an essential trace element for the human body, has become a prominent area of research in life sciences, with significant recent advances in understanding its role and the discovery of novel selenoproteins in human health. With growing global awareness of health and wellness, the demand for Se-enriched therapeutics, health supplements, and functional foods is expected to increase. Understanding the synthesis of selenoproteins has garnered significant attention, as Se is uniquely incorporated into proteins. Recently, selenoproteins from species across all domains of life have been characterized for their various health functions. Significant advances in life sciences have emphasized the importance of Se, particularly its incorporation into selenoproteins, where SeC is incorporated at active sites through the UGA codon. This co-translational insertion of SeC, first characterized in *Escherichia coli* and later confirmed in mammalian cells, remains a subject of intense research due to its complexity. Selenoproteins have a broad range of biological activities that contribute to cellular homeostasis, such as neutralizing ROS to mitigate oxidative stress. Excessive ROS can damage cells and tissues, leading to various diseases. Consequently, addressing Se deficiencies and exploring the potential benefits of Se supplementation for disease prevention are critical research areas. Emerging Se-enriched delivery systems, which combine Se with bioactive molecules, show the potential to enhance the bioavailability and therapeutic efficacy of Se. High-quality food-based Se supplements, have great potential to improve public health, offering a more sustainable supplementation approach than inorganic Se forms. Despite these advances, much is still to be learned about the structures and precise roles of certain selenoproteins. Further research is needed to elucidate their mechanisms of action, uncover their functional diversity, and explore their potential integration into functional foods aimed at improving health outcomes.

## Figures and Tables

**Table 1:** Selenoproteins, abbreviations, organs, and functions.

Selenoprotein	Abbreviation	Organs	Functions	Refs.
Glutathione peroxidase 1	GPx1	Highly expressed in the kidneys, liver, lungs, and erythrocytes	Antioxidants decrease cellular H_2_O_2_ and suppress the ability of retroviral virulence to cause mutations in viruses	[77,78]
Glutathione peroxidase 2	GPx2	Mostly found in the human liver and gastrointestinal tissues	Antioxidant activity lowers gastrointestinal peroxide, protects against oxidative damage and anti-apoptotic properties in the colon, and maintains the integrity of the intestinal mucosa	[79,80]
Glutathione peroxidase 3	GPx3	Mostly in plasma and extracellular fluid; additionally found in the GI tract, placenta, liver, kidneys, breasts, heart, and male reproductive system	Decreases peroxide levels in the blood and increases antioxidant levels in the plasma, protects the thyroid gland against thyrocyte hydrogen peroxide, and minimizes lipid hydroperoxides	[81,82]
Glutathione peroxidase 4	GPx4	Highly apparent in the mitochondria, testes, cytosol, and nuclei of cells	An antioxidant in membranes, lipid hydroperoxide detoxification functions in sperm as a structural protein and is also implicated in apoptosis; has an influence on male fertility	[83,84]
Thioredoxin reductase 1	TrxR1	Cytoplasm; intracellular content in the nuclei and cytosol	Decreases thioredoxin, regulates antioxidant activity, transcription factors, cell proliferation, and apoptosis; maintains redox balance; and facilitates DNA synthesis	[85,86]
Thioredoxin reductase 2	TrxR2	Widespread and located in the mitochondria	Regulates modulation of transcription factors, management of mitochondria-mediated cell death, preservation of mitochondrial integrity, detoxification of aldehydes, and support of protein synthesis and folding	[87]
Thioredoxin-glutathione reductase	TrxR3	Particularly in testes, also in mitochondria	Antioxidants, particularly within the reproductive system, essential for sperm development and maturation, are implicated in the pathophysiology of inflammatory bowel diseases, such as Crohn’s disease and ulcerative colitis	[88]
Iodothyronine deiodinase 1	DIO1	Primarily found in brown fat, thyroid, kidneys, and liver	Essential for systemic active thyroid hormone level production of active T3 cell hormones in thyroid and peripheral tissues	[89,90]
Iodothyronine deiodinase 2	DIO2	Endoplasmic reticulum membrane expressed in the central nervous system, pituitary, brown adipose tissue, heart, and skeletal muscle	T3 biosynthesis in peripheral tissues and thyroid hormone activation	[91]
Iodothyronine deiodinase 3	DIO3	Present in the skin, cerebral brain, uterine, fetuses, and CNS	Dio3 uses SeC for selective deiodination of thyroid hormones, with regioselectivity driven by loop dynamics and halogen bonds. Cys168 plays a role in regenerates, prevents the fetus from being overexposed to T3; deactivates thyroid hormone, and converts T4 to reverse T3 (rT3)	[92,93]
Methionine-R-sulfoxide reductase B1	MSRB1	Cytosol and nuclei	Involved in antioxidant defense and oxidoreductase activity and contributes to the regulation of redox in several tissues	[94]
Selenoprotein F	SELENOF, SEP15	Liver, brain, kidney, testis, thyroid, lung, and T-cells	Potential function in ER quality regulation of protein folding	[95]
Selenoprotein H	SELENOH	Nuclei, brain, and muscle cells	Redox-sensitive protein that binds DNA, with possible involvement in the activation of genes related to glutathione production	[96]
Selenoprotein I	SELENOI	Nuclear localization	Suppresses ferroptosis and supports intestinal and anti-tumor health	[97]
Selenoprotein K	SELENOK	Endoplasmic reticulum, skeletal muscle, and heart	Boosts microglial migration and phagocytosis by elevating Ca^2+^ via IP_3_R_3_ upregulation	[98]
Selenoprotein M	SELENOM	Endoplasmic reticulum	Likely functions as an oxidoreductase, contributing to redox balance and cellular homeostasis, with potential links to disease mechanisms through its Trx motif	[99]
Selenoprotein N	SELENON	A transmembrane glycoprotein related to ER	Protects against insulin resistance and muscle dysfunction by regulating ER stress	[100]
Selenoprotein O	SELENOO	Mitochondria	It may be a potential prognostic biomarker in various cancers, influencing tumor immunity, the microenvironment, and metabolism	[101]
Selenoprotein P	SELENOP, SEPP1	Extracellular glycoprotein is mostly present in plasma and is significantly expressed in the testes, liver, and brain.	SELENOP primarily transports Se and acts as an antioxidant; it is a key supplier of plasma Se and a reliable biomarker of Se status Deficiency of SELENOP in mice causes infertility; SELENOP also regulates tissue Se levels, with its genotype influencing Se concentrations in breast cancer tumors	[102,103]
Selenoprotein S	SELENOS	Predominantly in the endoplasmic reticulum and plasma membrane	Induces ER stress apoptosis, eliminates misfolded proteins from the endoplasmic reticulum, and regulates inflammation, a therapeutic target for LGG.	[75,104]
Selenoprotein T	SELENOT	Ubiquitous	SELENOT protects against cisplatin-induced AKI by reducing oxidative stress and apoptosis, with Nox4 inhibition partially reversing the effects of SELENOT silencing; also regulates neuroendocrine secretion and Ca^2+^ homeostasis	[105]
Selenoprotein V	SELENOV	Adipose tissue	SELENOV regulates lipid metabolism and thermogenesis; its knockout increases body weight, fat mass, and lipogenesis and reduces thermogenesis while lowering OGT protein levels and activity in adipose tissue	[106,107]
Selenoprotein W	SELENOW	Cytosol, skeletal muscles, and prostate	Protects against muscle atrophy in sarcopenia; osteoclastogenic	[66,108]
Selenophosphate synthetase 2	SPS2	Cytosol and nuclei	Triggers the synthesis reaction of selenophosphate, is a Se donor in biological responses, and helps synthesize selenoproteins	[109]

## Data Availability

The datasets generated during and/or analyzed during the current study are available from the authors upon reasonable request.
